# Stability and Variability in Aesthetic Experience: A Review

**DOI:** 10.3389/fpsyg.2017.00143

**Published:** 2017-02-07

**Authors:** Thomas Jacobsen, Susan Beudt

**Affiliations:** Experimental Psychology Unit, Humanities and Social Sciences, Helmut-Schmidt-University/University of the Federal Armed Forces HamburgHamburg, Germany

**Keywords:** aesthetics, aesthetic experience, experimental aesthetics, art, cognitive schemata, scripts, attitudes, sensitive period

## Abstract

Based on psychophysics’ pragmatic dualism, we trace the cognitive neuroscience of stability and variability in aesthetic experience. With regard to different domains of aesthetic processing, we touch upon the relevance of cognitive schemata for aesthetic preference. Attitudes and preferences are explored in detail. Evolutionary constraints on attitude formation or schema generation are elucidated, just as the often seemingly arbitrary influences of social, societal, and cultural nature are. A particular focus is put on the concept of critical periods during an individual’s ontogenesis. The latter contrasting with changes of high frequency, such as fashion influences. Taken together, these analyses document the state of the art in the field and, potentially, highlight avenues for future research.

## Introduction

In aesthetics, we deal with a multitude of factors influencing our preferences, judgments, contemplation, appreciation, liking, and disliking. While we are searching for laws of aesthetics universal for mankind, there is also no accounting for taste. While *Homo sapiens* appears to be the only primate species capable of fully developed aesthetic processing, there is also no denying that evolution has an influence on our aesthetic appreciation. While history has demonstrated a wide variety of often seemingly arbitrary ideals of beauty, we, as psychologists, would still hope to be able to identify general underlying mental mechanisms. For the endeavor of tracing stability and variability in aesthetic processing in this article, we will adopt a framework for the psychology of aesthetics that uses several vantage points (Jacobsen, 2006, 2010a). For one, it is based on psychophysics pragmatic dualism, using the mind and brain perspectives. It also holds the view that all episodes of mental processing of art, that is all episodes of aesthetic appreciation for our concern, manifested as an interaction of person and situation variables modified by different content domains. Furthermore, it asserts that changes happen over time (diachronia) and that they take place for different reasons and on differing timescales. Some may happen fast due to rapid changing in social influences, as seen in fashion for instance, while others happen on extremely slow time scales, like changes due to evolutionary biology. While biological universals may have been with the modern man for more than 100,000 years, and would thus be considered very stable, the same anatomy provides for extremely individualized and highly diverse preferences in various domains of aesthetic appreciation.

Aesthetic appreciation entails the evaluation of sensations and perceptions against relevant concepts like the beautiful, the elegant, the harmonious, the melodious, the rhythmical, and the like. These concepts show relative stability over time, over individuals, as well as aesthetic domains. The concept of beauty is central to many domains of aesthetic appreciation. On the other hand, the conceptual structure shows relative variability as well. Rhythm and melody are important for music and poems, but less so for faces (Jacobsen et al., 2004; Istók et al., 2009; Augustin et al., 2012; Knoop et al., 2016). For the present review, we have selected two theoretical psychological notions as a basic structure of our analysis, the notion of attitude and the notion of schema or script. Both are long term memory representations that have been shown to influence, if not guide, aesthetic appreciation.

There were various attempts to attain a reasonable definition of the concept of attitudes (for an overview, see e.g., Riemer et al., 2014), but all share the notion of evaluation. For a variety of objects humans have acquired relatively stable “[…] summary evaluations […] ranging along a dimension […] from positive to negative” (Petty et al., 1997), so that in sum an attitude object can be linked with liking/disliking. Thus an individual who is about to aesthetically appreciate an (aesthetic) object, will approach it with its own attitudinal system. That system may, for instance, comprise evaluations of one’s own expertise with regard to the aesthetic domain or, more specifically, the genre at hand. If information about the creator of the aesthetic object is available, the aforementioned may be implicated with stored evaluations of various groups. In addition, the aesthetic object might address explicitly specific themes or induce associations with specific related issues (even if not intended by the creator) for which summarized evaluations are stored and automatically activated upon exposure with the object.

The schema concept has also been used and described in various ways, mostly based on conceptions suggested by Piaget (1926) and Bartlett (1932). According to Mandler (1984), “knowledge about an object or classes of objects, about an event or classes of events, about personality traits and social norms, can all be considered as small networks of information that become activated as we experience these things”. Thus, schemata are higher-order cognitive structures that are relevant to understand how old knowledge and past experiences interact with new information (Brewer and Nakamura, 1984). Referring again to a situation in which an object is aesthetically appreciated, the perceiver not only approaches the aesthetic object with a set of relevant attitudes, but also holds ready applicable schemata. Whereas attitudes often elicit reactions or responses toward the attitude objects, schemata provide frameworks which allow for making sense of the given situation or aesthetic object by adding missing details. Based upon the attributes and their interrelations which are stored for a particular schema, expectations are formed and missing information can be added. Depending on various aspects of the situation, for instance, the type of aesthetic object (e.g., static painting versus dynamic piece of performance art) or the context of the experience (e.g., private house versus museum versus laboratory), different (types of) schematic representations are activated. For aesthetic appreciation self-schemas and event schemas, which are commonly referred to as cognitive scripts, may be foremost important. A script holds information about “appropriate sequences of events in a particular context,” is composed of “slots and requirements about what can fill those slots,” and due to interconnections “what is in one slot, affects what can be in another” (Schank and Abelson, 1977). For instance, different scripts will be addressed during a museum visit and during a theater play. In sum, the activated set of attitudes, schemata and scripts upon exposure to an aesthetic object may then modulate aesthetic appreciation.

We trace the importance of these mental representations through different domains of aesthetic processing, while we investigate the variability or stability of either the mental representation itself or variability or stability of its contents. Attitudes and schemata/scripts are very frequently acquired implicitly. They are influenced unintentionally, or overlearned intentionally. Their flexibility or plasticity may change over time, may change over the course of ontogeny. Critical periods for either the acquisition or the change of an attitude or a schema may apply. It is possible that while the content of the script is bound to change on a regular, fixed basis and time scale, the script itself has been acquired for life and is thus stable.

There may be domains of aesthetic processing in which individual differences in aesthetic appreciation have to be accounted for in order to be able to identify underlying general mental mechanisms. For instance, the identification of the brain correlates of aesthetic judgment of beauty of novel, formal graphic patterns required the formal modeling of the participants’ individual judgments (Jacobsen et al., 2006). That is, while individual beauty ratings for the graphic material presented to the participants were highly heterogeneous, a common neural network subserving the process of aesthetic judgment could be identified.

This review is organized as follows. The first section addresses the role of schemata and scripts in the context of aesthetics. In the second section the role of attitudes and preferences in aesthetic appreciation is explored. This is done by first exploring developmental aspects (e.g., sensitive periods). Subsequently variability and stability of relevant attitudes and aesthetic preferences across different aesthetic domains are elaborated on the basis of respective research accounts.

## Schemata/Scripts

It is, most likely, safe to assume that we all command long-term memory representations of the schema and script type of various aesthetic domains, arts, and art genres. If we have not intended to acquire such a memory representation actively, we have implicitly acquired it.

Implicitly acquired, we most likely command a host of, in part, very diversified schemata and scripts. Besides prescribed actions or rather non-actions, like not touching exhibits in a gallery or a museum, schemata and scripts also entail and prescribe affective responses to art. Taking the conceptual structure of the aesthetics of literature as a starting point, the study by Knoop et al. (2016) shows that comedies as a literary genre prescribes more funny cognition than tragic, for instance, while plays, in general, including drama, are prescribed as tragic, dramatic, and also sad. The data show that, most likely, theater goers as well as readers of drama or comedy command a script of the reception of such a play that also includes elements of an affect script. Going to the cinema planning to watch a sad movie, entails elements of emotion regulation. We know that there will be sad content. Studies by Hanich et al. (2014) and Wassiliwizky et al. (2015) demonstrated that a very important emotional response to sad movies is the emotion of being moved. Being emotionally moved by the movie one watches most likely is an element of emotion regulation mediated by the art reception script including elements of an effect script. Scripts of this rather general type appear to be rather stable, although there is no empirical study on a critical period of acquisition and or the stability of these scripts overtime.

Just as other social conventions are acquired over a number of experiences, art schemata of different domains are frequently acquired implicitly. Attending a concert of classical music entails being quietly seated, avoiding making noises, not getting up to dance, not talking to fellow listeners, spending applause only in the right moments in time, and so on and so forth. Attending the showing of a theater play conventionally entails being quietly seated avoiding making noises, not talking to one’s neighbor, not getting up to dance, and spending applause only in the right moments. This does, of course, not mean that such a script cannot be broken. As it often occurs in modern theater, audience may be urged to participate, like actually getting up to dance, for instance. In such a moment, viewers may feel insecure and uncomfortable, if not used to participate in things like that. This little anecdote demonstrates that implicitly acquired scripts and schemata may very well have the power to shape our experience, to be real. It also shows that scripts and schemata may contain certain default slots that guide our experience when filled. For instance, in the above mentioned examples of classic concerts and theater plays, the audience is at a safe distance to the performance. In the conventional version of the script, audience participation clearly is not called for. Thus, the members of the audience do not have to be afraid of being exposed to other members. Rather, one can safely assume to be left in the exclusively receptive role. This fact may foster focuses on individual subjective feeling, self-referential appreciation and evaluation of sensation and perception.

Some schemata may actually be quite diversified and detailed, even on the bases of implicit acquisition. Art schemata and scripts are constituted in a social fashion. This means they are subject to change on the bases of social and cultural change. Therefore, stability and variability of art schemata and scripts hinge on cultural and social factors. On the other hand, some very basic schemata may even have evolutionary underpinnings. An example may be the basic narrative scheme, making minimum requirements for a narrative plot. The art form for poetry, for instance, may have characteristics that speak to a co-evolution of our language and memory faculties. The more evolutionary grounding an arts schema or art script has, the less likely it should be subject to change, and thus be very stable.

The notion of scripts for the reception of performing arts as introduced by the affect script example above, most likely, extends to various narrative and/or performing art forms. To date, we do not know of systematic studies on the acquisition and the ex-act design of these scripts.

## Attitudes and Preferences

Long-term memory representations such as attitudes are perceived as sets of evaluations stored for an object and ranging over a positive – negative dimension (Petty et al., 1994). A common notion is that attitudes entail a knowledge component, a valence aspect and a behavioral tendency. Differences of their underlying structure are thought to influence their strength, i.e., their persistence over time, resistance against counterpersuasion and thus the degree of their influence on judgment and behavior. Getting activated automatically, those evaluations facilitate a quick assessment of the everyday object being processed (for a review, see Petty et al., 1997).

Representing an important modulator of aesthetic experiences, attitudes may often override other effects for which influences on aesthetic appreciation have been demonstrated (i.e., fluency, familiarity, mere exposure). Theoretical accounts for this modulation are still scarce. Some attention has been drawn to this issue within the framework of automatic and controlled processes of aesthetic appreciation provided by Jacobsen (2010b), in which memory systems operating at different levels of aesthetic appreciation are reviewed.

When stimuli are being accommodated in the perceiver’s attitude system, the latter may have a profound influence on the aesthetic appreciation of these stimuli (see Ritterfeld, 2002, for a seminal study or Istók et al., 2013). In fact, attitudinal influences could even lead to outright memory-based judgments of beauty or other important concepts (Jacobsen et al., 2004; Istók et al., 2009; Augustin et al., 2012; Knoop et al., 2016), instead of proper aesthetic processing. Hence, some past research endeavors in the field considered attitudinal influences as confounding factors that needed to be reliably excluded, for instance by using novel stimuli (visual stimuli: e.g., Jacobsen and Höfel, 2002, 2003; Höfel and Jacobsen, 2003, 2007a,b; Jacobsen, 2004a; music stimuli: e.g., Brattico et al., 2010; Kornysheva et al., 2010; Istók et al., 2013) that could less likely be integrated automatically in the perceiver’s attitude system on grounds of knowledge and expertise.

One common way to gauge the automatic activation of attitudes is the application of the implicit association test (IAT), which allows an exploration of the unconscious use of the attitudinal memory system (e.g., Fazio and Olson, 2003). By now this approach has been adopted in a few studies within aesthetic domains such as the human body (Ahern et al., 2008), visual art and architecture (Mastandrea et al., 2011), and design objects (Mastandrea and Maricchiolo, 2014). We will now explore findings regarding sensitive periods for the development of attitudes relevant to aesthetic contemplation and we will try to relate those findings to the development of aesthetic preferences for different domains of aesthetic appreciation.

### Acquisition and Sensitive Period of Attitudes and Aesthetic Preferences

Appreciation of art and aesthetics are often considered as being unique for the human species (e.g., Dobzhansky, 1962). During ontogenesis of both the human perceptual system and the human conceptual system, foundations are laid for the capacity of aesthetic evaluation and thereby as well for the formation of taste. Several aspects support this notion.

Regarding the development of visual perception, among those aspects is, for instance, the existence of ocular dominance columns (Hubel and Wiesel, 1962), for which a critical period (postnatal up to 5 and more years of age) for their experience-dependent neuroplasticity has been widely investigated (for a review, see e.g., Hooks and Chen, 2007). Another aspect refers to functional specialization of individual neurons as well as of neural circuits for the processing of, for instance, single features (like color or motion direction; see e.g., Zeki et al., 1991) or complex shapes and objects (like faces, substantially processed by the fusiform face area; Kanwisher et al., 1997; Kanwisher and Yovel, 2006). The development of face processing sensitivity has been investigated in a study with subjects, who have been blind from birth but who had their vision restored via cataract surgery (Röder et al., 2013). Due to the varied time periods of blindness among subjects, identification of a closer defined sensitive period has not been possible, though the results could demonstrate that such a period exists. As to sensitive periods for the development of other characteristics of the visual system, different time periods seem to exist, in that, for instance, the development of normal visual acuity and peripheral light sensitivity are impaired when visual deprivation begins at 6 months after birth (review by Lewis and Maurer, 2005). Interestingly, this does not apply for global motion sensitivity for which impairment could only be shown when visual deprivation began near birth (Lewis and Maurer, 2005).

Within the limits of this manuscript, we do not aspire to give an ample overview of the findings regarding sensitizing periods for all perceptual qualities. Preliminary to reviewing findings regarding the development of aesthetic preferences and attitudes toward musical stimuli, we will shortly address sensitive periods for the auditory domain. Similar to the development of the visual perceptual system, for auditory perception the early years after birth are crucial for developing normal auditory feature processing (for reviews, see e.g., Trainor, 2005; Howard-Jones et al., 2012). For instance, the neonate brain’s sensitivity to discriminate acoustic differences is initially very high, before it learns to organize auditory input according to distinctive features and to belonging to certain categories while learning to ignore more and more insignificant variability of sounds (for a review, see Kral, 2013). Besides being obviously crucial for language learning (for a review, e.g., Kuhl, 2010), this initial sensitive period is also relevant to musical learning (Penhune, 2011). Young infants are able to discriminate acoustic properties (e.g., timing and pitch, Trehub, 2003) and they are sensitive to processing rhythm, melody, aspects of harmony (Trehub, 2006; Trehub et al., 1997), which is relevant for music processing and therefore for later musical preferences (for a review, see Nieminen et al., 2011).

Whether musical preferences are biologically determined or acquired through exposure is still being debated. Studies investigating musical preferences in neonates and young infants indicate that sensory processing of basic musical properties, along with core liking of those properties (like preference for consonance: Perani et al., 2010), occur early in child development (Nieminen et al., 2011, 2012).

The development of a distinctive musical taste which characterizes an individual has been outlined by Brattico et al. (2013). In their neuro-cognitive model of the aesthetic processing of music they proposed that musical taste results from a long-term set of preferences, attitudes, values and aesthetic judgments and those in turn should be influenced by individual characteristics such as age, gender, social status, and personality. Whether listeners arrive at consciously liking or disliking a musical piece should be determined by an interaction of their musical taste and their context variables (mood, attention, social attitudes). The authors described this as building a feedback loop which should be more pliable within early adulthood if compared to infancy or adulthood (Brattico et al., 2013). In young listeners (daycare and elementary school) music preferences have been shown to be strongly affected by social factors such as preferences of parents and siblings (Roulston, 2006) and for 7- to 8-year-old children exposure to gender stereotypic role models seems to be relevant for choosing a musical instrument (Harrison and O’Neill, 2000). Late adolescence and early adulthood have been linked to individual’s increased musical genre exploration, which is in line with the open-earedness hypothesis (LeBlanc et al., 1996; Hargreaves and North, 2010). According to the open-earedness hypothesis younger children show more tolerance toward musical styles, which are categorized as unconventional by adults, possibly as a result of being less “adapted to” normative standards with regard to what is considered as good and bad taste in the given culture. Exploring various musical genres in this developmental stage is thought to help shape and crystallize musical taste.

Lately it is discussed, that adolescence may provide also periods for sensitization, mainly concerning higher-order processes (Howard-Jones et al., 2012; Fuhrmann et al., 2015). It was also suggested, that for bi-directional transfer of skills between language and musical processing there may exist a sensitive period as well (White et al., 2013).

### Variability and Stability of Attitudes and Aesthetic Preferences

The notion of a universal in aesthetic appreciation constitutes the strongest variant of stability. Historically and culturally invariant, its traces can be observed in individual behavior. In a psychophysical sense, adopting the mind-brain perspective, taste can be considered a long-term representation that is more or less changing, or the respective bodily substrate. Furthermore, taste also comprises a more generalized element when regarding the scope of a genre or style (for musical domain, see e.g., Istók et al., 2013).

Taste in general is subject to change which happens at different time scales. In fashion, taste once was expected to adapt on a 6 months’ cycle. By now this has been taken to the extreme, anecdotally in the context of fast fashion cheaply imitating fashion trends and involving (in the case of some fashion brands) up to 6 mid-seasonal trends per year. There is most likely, although evidence is still scarce, highly stable taste based on ontogenetic acquisition during critical periods (as depicted in Chapter 2.1). On the other extreme, there are species universals that are expected to be stable, or at best culturally modified. Research investigating universal, biologically based aesthetic tendencies and their shaping by cultural and historical influences (see ipsichronia vantage point, Jacobsen, 2006) is scarce so far (for an overview on cross-cultural differences and similarities regarding attractiveness of faces or body features, see Little et al., 2011).

One factor that is regarded as a standard in aesthetic appreciation shared across cultures is symmetry, which seems to be one of the biologically based aspects of beauty. Though within culture perspective there exist accounts of more variability across aesthetic domains. For the aesthetics of faces similar aesthetic tendencies across different cultures have been found (Little et al., 2007). Within the domain of the aesthetics of architecture and urban space, a more differential picture of the importance of symmetry has been shown. Investigating the link between the visual, spatial properties of complex streetscapes and their aesthetic judgments, Weber et al. (2008) found that visual regularity informed by parameters of symmetry and uniform arrangements of lateral boundaries in the streetscape photographs seems to be a primary factor in beauty preferences. Though, analyses controlling for professionalism in architecture and urban planning, indicated that symmetry/asymmetry seem to be differentially important factors for beholders of different expertise levels.

Along with symmetry, the complexity factor of an aesthetic stimulus has been discussed as a strong predictor of aesthetic judgment (Jacobsen and Höfel, 2001). The effects of both factors on aesthetic judgment seem to be quite robust, even across different stimuli, participants and contexts (Tinio and Leder, 2009). Interestingly, more recent accounts investigating familiarization with stimuli have revealed findings that are inconsistent with study results on mere-exposure (e.g., Zajonc, 1968). Thus, the robust effects of symmetry and complexity seem to be modifiable by massive familiarization (Tinio and Leder, 2009), which has been thought of eliciting a subsequent preference of novel stimuli (Biederman and Vessel, 2006).

A recent comparative eye-tracking study across cultures and species showed that humans with a different cultural background (Namibian hunter-gatherers and German town dwellers) both showed longer fixation on symmetric abstract graphic stimuli (adopted from Jacobsen and Höfel, 2002), irrespective of pattern complexity, whereas non-human primates (orangutans) did not differentiate between symmetric and non-symmetric patterns but showed faster and broader scanning of the presented screen (Mühlenbeck et al., 2016). Comparison of aesthetic evaluations between both human groups revealed that aesthetic preferences were in accordance with fixation preference only within the German subject group, but not in the Namibian group.

One of the strongest contenders for a universal law of beauty has been considered the Golden Section. In “Vorschule der Ästhetik,” Fechner (1876) illustrated the role of the Golden Section Hypothesis in determining aesthetic preferences. Importantly, there are empirical accounts both supporting and contradicting (for an overview, see e.g., Konečni, 2012) the idea of the fundamentally important golden ratio, the latter accounts pointing at a high susceptibility of the effect to studies’ methodological differences (Höge, 1995).

Another theoretical aspect which has been thought of as constituting a universal is the correspondence of basic colors and forms. If such a correspondence exists, we would expect it to be highly stable. The desire to find basic, generally applicable principles for art and design in early 20th century gave rise to theorizing about a universal and ideal visual language, and resulted in the color-form assignments developed by Wassily Kandinsky (Jacobsen, 2002, 2004b). Thenceforward Kandinsky’s assigned yellow triangle, red square and blue circle became a well-known theme in the Bauhaus school of design. Remarkably, assessing color-form assignments in those a study with non-artist students, Jacobsen (2002) could show that Kandinsky’s combinations were the least preferred. In both tasks (mere color-form correspondence vs. aesthetic correspondence), about half of the participants showed clear and stable assignments (red to triangle, blue to square and yellow to circle) and stated world knowledge associations in the rationale for their choice. Yet, another study with visual arts experts demonstrated different color-form assignments (Jacobsen and Wolsdorff, 2007). Those results led to the notion that a multitude of factors (i.e., world knowledge, education, and historical change, as well as individual, group-specific, and societal leitmotifs) influence individual color-form preferences and that the Bauhaus combinations were historically/culturally determined (Jacobsen and Wolsdorff, 2007).

Another combination of basic perceptual qualities such as color and sound and their corresponding approach-avoidance patterns have been investigated recently in a comparative eye-tracking study on human and non-human primate species (Mühlenbeck et al., 2015). Regarding biases for and avoidance of colors in human subjects, this study only partially supported previous findings (i.e., avoidance of yellow). Eye-tracking data for non-human primates (orangutans) did not indicate clear gaze duration biases or avoidance for color at all. Other than hypothesized, no modulation of color approach-avoidance patterns by consonant and dissonant auditory stimuli could be found for both human and non-human subjects. The authors concluded that in specific contexts color preferences could be independent of perceiving auditory stimuli and that the lack of specific color biases and color avoidances in both subject groups neither support nor disagree with hypotheses regarding the advantageous evolution of trichromatic color vision.

Among all of the contenders for aesthetic universals one of the most realized across cultures seems to be the factor of skin quality. Apart from a few exceptional examples regarding beauty related skin condition (e.g., decorative scarring in African tribes, facial tattooing in women of Myanmar’s chin tribe), a flawless and even skin texture as well as homogeneity of skin color seem to be primary determinants of perceived attractiveness (e.g., Symons, 1995; Fink et al., 2001, 2006). This influence of skin quality has been explained in terms of perceived higher health condition, reproductive ability and youth in general.

#### Visual Art and Photography

Research on aesthetic preference in visual arts has so far focused on group differences in order to find general rules. However, individual differences of aesthetic judgments have been revealed in few different domains, one of them being that of visual art (e.g., Jacobsen and Höfel, 2002; Jacobsen, 2004a).

Höfel and Jacobsen (2003) could demonstrate that beauty judgments of novel, formal graphic patterns (taken from Jacobsen and Höfel, 2002) made by undergraduate students with no expert knowledge in fine arts were changing within several weeks, thereby showing low stability of individual judgments of aesthetic value. Following up on this finding, Jacobsen (2004a) investigated individual differences in beauty judgments of another set of novel, formal graphic patterns, comparing individual performance and group models for ratings and rankings made by a homogeneous group of non-artist college students. It was found that the group model, based on data averages, could only account for half of the participants’ judgments, whereas better representation of the data was achieved by the individual models. Beauty judgments of a number of participants even resulted in contrary ratings and rankings, being intra-individual consistent.

In those studies, which used novel graphic patterns, the individual differences in aesthetic judgment of beauty would have led to null results if prescriptively classified objects would have been grouped for the analysis. Rather, individual judgment policies have been captured using methods of judgment analysis. This way, different subsets of stimuli have contributed to conditions in different participants. In view of the above findings, which indicated manifest individual differences in aesthetic preferences, it may be essential to group judgment categories rather than object categories. As implemented by Jacobsen et al. (2006), identifying brain correlates of aesthetic judgments of beauty using these graphic patterns was only possible by employing this approach.

Few studies have also shown individual differences for color preferences (McManus et al., 1981; Whitfield, 1984). Those preferences appear to depend to a great extent on the context, specifically on the object to be colored. Regarding the study by Whitfield (1984), participants showed substantial individual differences of color preferences for walls of a domestic interior furnished in one of three styles (Modern, Georgian, Art Nouveau) when ask to perform corresponding appropriateness rankings of given color samples.

Since at least conceptual art emerged in the middle of the 20th century, it stands to reason that defining art requires reference to its context (Gartus et al., 2015). Contextual influences on emotional responses toward different kinds of artworks (abstract paintings vs. graffiti artworks) were demonstrated in a study by Gartus and Leder (2014). They presented both types of artworks in one of two contexts (museum vs. natural street scene) and showed that participants with a positive attitude toward graffiti art even elicited more positive evaluations of those artworks in a street context (compared to a museum context). The authors concluded that individual attitudes toward art styles modulated contextual effects on the evaluation of artworks. Following up on those results, Gartus et al. (2015) investigated specifically which effects context could have on perception and appreciation of an artwork. Again they found that aesthetic appreciation can be significantly influenced by an artworks context and that some of those effects depend on the style of artworks and some result from an interaction of context and individual interest in art styles.

In another study, implicit aesthetic preferences for figurative as opposed to abstract art styles have been shown (Mastandrea et al., 2011), involving shorter response latencies when participants (non-experts in arts) associated positive (vs. negative) words with figurative (vs. abstract) art.

As to the aesthetics of photography, to date few studies have investigated specific attributes that might account for favoring one (artistic) photograph over another. Often photographs have been used as a technical means to investigate other questions within experimental aesthetics and most experimental approaches, directed at photography itself, focused more on the perceptual than on aesthetic processing of photographs. Certainly, some of the attributes which are discussed as being relevant for aesthetic preference in visual and fine arts might be relevant for the aesthetics of photographs as well. The few empirical findings suggest that features determining image quality seem to be important for the liking of a photograph. In two studies, Tinio and Leder (2009) and Tinio et al. (2011) used images of natural and human-made scenes for which they manipulated certain image features (systematic degradations of contrast, grain size, and sharpness/focus). Depending on the type of scene (stronger effects in human-made as compared to natural scenes), they found that certain degradations and their combinations (higher contrast, less grain, better focus) had more impact on aesthetic preference than others. Furthermore, their results showed an additive effect for feature degradations (an image was less liked the more degradations it was subjected to).

Composition plays a role in a lot of stimuli that can be experienced aesthetically. To examine compositional effects experimentally, a few studies focused for instance on cropping of photographs and its determinants (McManus et al., 2011; Abeln et al., 2016). It was observed, that cropping ability differed substantially between individuals and that cropping is driven more by top-down knowledge of objects (McManus et al., 2011) and that saliency and balancing of different salience regions seems to play an important role for aesthetic appreciation and preference (Abeln et al., 2016). Regarding image attributes that are traditionally discussed within visual aesthetics, such as the rule of thirds and the golden section, the rare studies investigating those in the context of photographs did not reveal a clear pattern of findings yet. Surprisingly a lot of factors which may influence the aesthetic appreciation of photographs have not been or have been only insufficiently investigated yet. To the best of our knowledge, this applies as well to the development of taste in art photography.

#### Music

Up to date neuro-cognitive models of human mental facilities and sensory modalities are quite sophisticated. In view of substantial differences in the mental processing architecture across domains like vision and audition, it stands to reason that there are domain-specific characteristics of music processing which have to be taken into account in addition to modality- and domain-independent processing stages. This has been implemented in the neuro-cognitive model proposed by Brattico et al. (2013), in which domain-specificity is considered an important aspect in the aesthetic processing of music.

In contrast to other aesthetic domains like dance, language, or architecture, research on the aesthetic appreciation in the musical content domain has been progressed notably within the last decade. The respective model by Brattico et al. (2013) now constitutes a relevant reference point for present-day research. According to their elaborations, initial processing stages (feature analysis, multisensory integration, top-down cognitive processing) in the course of an aesthetic appreciation of a musical stimulus, are common among individuals which share the same background of musical culture. Subsequent stages are characterized by controlled cross-modal neural processes underlying intermingled cognitive, affective, and decisional processes which result in aesthetic emotions, aesthetic judgments and conscious liking. Although often used as synonyms, the authors differentiate “liking”, which is considered as a more ongoing process, from “preferences.” Preference for a musical style or genre is regarded as stable over time and has been shown to be determined for instance by social factors (peer groups, musical taste of parents and siblings, especially during adolescence; Roulston, 2006) and an individual’s personality (e.g., Rentfrow and Gosling, 2003; North, 2010). Research on musical genre preferences have to take into account difficulties in defining genres and the respective interindividual differences as well as the development of genres and of their sociocultural meaning over time, which might limit the validity of assessed genre preferences. Other than for musical genres, preferences for a musical instrument seem to be more context-sensitive, at least in children, whose choices of musical instruments have been shown to be susceptible to social schemata such as gender stereotypes (Harrison and O’Neill, 2000).

Research examining those factors that are assumed to account for a certain variability of musical liking has focused, e.g., on mood (for a review on general mood influences, see Konečni, 2010; on sad music and mood congruency, see Sachs et al., 2015), attention and intentionality (for an overview, see Brattico et al., 2013). As summarized by Brattico et al. (2013), a certain variability of the aesthetic experience of music is brought about by internal (e.g., expertise, mood, attitudes) and external context factors (environment, peers, task at hand). One explanation for an influence of expertise on musical preferences (as shown, e.g., by Müller et al., 2010; Brattico et al., 2016) seems to be that expertise provides specific prototypes (Leder et al., 2004). Prototypicality has been proposed to be preferred (Martindale, 1988) and for the domain of music it has been shown to positively influence preferences (Smith and Melara, 1990; Brattico et al., 2009b).

Another interesting study showed that lyrics (as another external factor) have differential effects in aesthetic experience of happy and sad music, that is instrumental cues were shown to be more important in happy music (with stronger positive emotions for happy music without lyrics), both happy and sad musical pieces without lyrics were liked more than those with lyrics, and lyrical cues seemed to be more important for defining and inducing sad emotions (Brattico et al., 2011). Attribution of a value (positive or negative) to music is observable during an individual’s life span and across cultures (Brattico et al., 2009a). On a neural level, basic emotion perception was associated with structures being spatially separate from motivational and evaluative processes and seems to be processed earlier than the latter (Brattico et al., 2016). This implicit appraisal of the affective state which is induced by a musical piece seems to build the basis for aesthetic enjoyment, conscious liking and various judgments linked to the aesthetic episodes (e.g., beauty judgment; Brattico et al., 2016). Sound features like complexity or sensory dissonance can be linked to more stable aspects of aesthetic experiences and to domain general perceptual preferences across different musical and auditory domains (Brattico et al., 2009a). More specifically temporally or spectrally complexity is preferred over less complexity (Tervaniemi et al., 2000; Brattico et al., 2001; Fujioka et al., 2004) whereas sensory dissonance is shown to be related to avoidance behavior of individuals as early as 2 months old (Trainor et al., 2002). It has been proposed that the listening context (everyday situation vs. aesthetic context like a concert hall) might either induce solely basic emotions (everyday situations) or special aesthetic emotions such as aesthetic enjoyment, awe, nostalgia or being moved (Brattico and Pearce, 2013).

The numerous factors and their netting of complex interactions, which are thought to influence the aesthetic experience of music, seem to account for a high variance of subjective musical preferences (Brattico and Jacobsen, 2009).

#### Language and Literature

A recent study shed light on the conceptual structure underlying the aesthetics of literature, using a verbal association task (Knoop et al., 2016). The conceptual representations were also investigated specifically for the aesthetics of literary forms and genres (short stories, novels, poems, comedies, plays). Comparison to findings for other conceptual structures in the domains of visual art and music revealed the greatest overlap between literature and music. Particularly, as beauty was named as one of the most frequent terms for almost all domains investigated, it seems to be an important category for non-expert conceptualization of aesthetics (Jacobsen et al., 2004; Istók et al., 2009; Augustin et al., 2012; Knoop et al., 2016). Though, Knoop et al. (2016) derived from their findings a different role of the term beautiful when literature is denoted. More specific, rather prosodic language qualities and feelings (or moods or atmospheres) described as romantic or poetic seem to underlie an attribution of literary texts as beautiful. For overall aesthetics of literature, suspenseful was another prevalent term. Apart from that, the most frequent labels varied between literary subgenres. In addition, there was a predominance of evaluative (compared to few descriptive) terms, as previously found for the aesthetics of objects (Jacobsen et al., 2004). Knoop et al. (2016) suggested that within aesthetic conceptualization even the apparently purely descriptive labels may comprise an evaluative dimension. For instance, rhythmic was among their top listed music-related terms for poetry (besides harmonious and melodious) and in a previous study regularity in poems was associated with positive aesthetic liking judgments (Obermeier et al., 2013). There are also empirical accounts that indicate that even when not explicitly instructed to do so, spontaneous evaluation takes place during silent reading (Bohrn et al., 2013).

It was discussed, that it was not possible to derive to which extent the assessed conceptual representations for the aesthetics of literature (and subgenres) arose by reason of acquired theoretical concepts or by the participant’s own experience with the contemplated subgenres (Knoop et al., 2016). Thus, although there are rare empirical endeavors yet which investigated literary aesthetic preferences, their degree of stability and potential influencing factors accounting for a certain context-dependent variability, the presented information regarding the concepts associated with aesthetic attribution in the domain of literature contribute to a deeper understanding of how aesthetic appeal dimensions are perceived and conceptualized across aesthetic domains.

#### Dance

It has been suggested that watching a dance movement involves a simulation of this movement using the same neural network that is active while executing it (Calvo-Merino et al., 2005). It might be reasonable or even essential to include considerations of more basic movements in order to tackle the aesthetics of dance. Orgs et al. (2013) have broken down the aesthetic experience into the investigation of aesthetic effects of three levels of human movement representations. Their findings indicate that aesthetic preferences can be influenced by body postures, movement continuation (“good” vs. “bad”) and choreographic structure. The finding, that initial familiarization with asymmetrical sequences increased later liking of such asymmetrical sequences, also suggest a structural mere exposure effect.

Drawing upon the discovery of mirror neurons and the concept of embodied simulation, it has been argued that the activation of universal mechanisms encompassing the simulation of emotions, actions and bodily sensations, is a crucial part of the observer’s aesthetic response to those stimuli (Freedberg and Gallese, 2007). According to them, such embodied resonance in response to an image can also arise if emotions, actions and bodily sensations are merely implied by the physical traces of artwork production and even in the absence of an overt emotional component. Cross et al. (2011) suggested that the above conceptualization could be further applied to more dynamic forms of art and they reasoned that embodied simulation may influence the aesthetic experience of dance performances. In their fMRI study, they had participants judge how well they think they were physically able to reproduce a range of dance movements (performed by professional ballet dancers) and how much they liked those movements. Results indicated a preference for watching more difficult movements (associated with a higher activation of bilateral occipitotemporal and right inferior parietal portions of the action observation network (AON)). It seems that increased aesthetic ratings for dance movements that have been mentally simulated or even physically practiced do not result from mere exposure or familiarity effects, as suggested by findings of later training studies (Kirsch et al., 2013, 2015). The findings by Kirsch et al. (2013) didn’t show increased enjoyment of dance movements in the view and music, or music-only conditions, suggesting that actual performance of dance movements additionally contributes to the aesthetic experience of dance. Cross (2015) described the interaction of embodiment and aesthetic experience as resembling a U-shaped function. This perspective might partially explain the high preferences for familiar and likely physically reproducible dance movements as well as for those dance movements which are spectacular, novel and not physically reproducible.

In their studies Stevens et al. (2009) used personal digital assistants (PDAs) to assess psychophysiological data of participants viewing a dance performance. In contrast to emotional arousal, which has been higher during more important dance parts (according to choreographer and experimenter ratings), no correlations with specific dance parts have been found for emotional valence. A wide variety of individual’s interpretation of and experience with dance performances has been discussed as a potential factor that led to the latter result.

#### Architecture and Environmental/Urban Space

Consideration of aesthetic aspects is part of architectural creation itself, thus both are closely linked and, perhaps to a similar extent like in the domain of visual arts, widely discussed within philosophical, architectural, and psychological frames. Research endeavors in the field have tried to tackle general principles for an influence of physical features on the evaluation of aesthetic experience of buildings and their environments (Nasar, 1994). As reviewed by Nasar (1994), three kinds of aesthetic variables became apparent on that score, that is formal variables (factors such as complexity, enclosure and order), symbolic variables (e.g., style) and schema (e.g., typicality). The author’s analyses of empirical accounts derive advice for design reviewers seeking different evaluative responses based on combinations of the above listed feature characteristics. Besides considerations of aesthetically relevant aspects of buildings themselves, features of a building’s context, ranging from closely outlined surroundings to vast streetscapes encompassing several other objects and their features within urban space (e.g., parks, stations, railways, space between objects), are also regarded in terms of their influence on aesthetic appreciation and should be addressed just as well within experimental aesthetics. Selecting habitats and creating shelter are arguably very old human behavior.

First, research findings fitting the more narrowly drafted architectural domain will be reviewed before addressing the aesthetics of urban space in the second part of this paragraph.

Gifford et al. (2000) had architects and laypersons aesthetically judge their global assessment (ranging on a 10-point scale from terrible to excellent architecture) of modern office buildings. Additionally, assessed pleasure and arousal ratings for those building photographs have been related to objective features of the buildings. Global assessments of the buildings have been found to be based on elicited pleasure but not on elicited arousal for both groups. The authors found, that aesthetic evaluations of experts and laypersons were almost unrelated and they concluded that this might be due to the fact, that both groups based their emotional assessments on different groups of objective features of the building’s exterior. For expert participants pleasure regarding an office building has been related to objective features such as more metal cladding, more railing, and fewer arches. Those physical features seemed to signify more complex ideas for architects, such as prototypicality and richness of material. In a different way, layperson’s pleasure ratings were not derived from even one of the assessed objective building features. This has been explained in the context of layperson’s greater within-group heterogeneity of pleasure ratings, the latter being more subject to various influences in laypersons in contrast to expert ratings of architecture. However, an additional interesting result has been, that none of the three assessed contextual variables (visible amount of landscaping and roads, human presence/activity evidence) had a significant influence on global assessments, pleasure, and arousal ratings, which indicated little impact of non-building context.

Another study by Marković and Alfirević (2015) compared artist’s and non-artist’s aesthetic judgments of architectural expressiveness of photographed buildings. Two clusters of architectural objects have been obtained within analyses. A choleric expressiveness cluster including objects rated high on the factors aggressiveness and color and low on the factor regularity, and the phlegmatic expressiveness cluster encompassing architectural examples with low ratings on Aggressiveness and Color, and high ratings on Regularity. The authors associated objects of the former choleric cluster with expressionistic and the latter phlegmatic cluster objects with minimalist architectural character. Interestingly, further analyses suggested that although the two groups of architectural objects differed regarding their allocated expressiveness, aesthetic pleasure has been rated virtually equal for both across participants. Expertise related differences in aesthetic preferences (experts rated objects included in the phlegmatic cluster as more aesthetic, whereas non-experts judged choleric cluster objects as more aesthetic) have been interpreted in terms of processing fluency and Berlyne’s (1971) optimal level of arousal. Marković and Alfirević referred to architects (meaning experts, as against non-experts) as being more responsive to new experiences and as preferring abstract and less comprehensible content (compared to non-experts’ preferences of clearer and pleasant content; empirical accounts by Rawlings, 2000, 2003). Given that, they argued that the phlegmatic cluster might have encompassed objects that have been experienced as more interesting and thus more pleasant for experts, whereas the same objects seemed to have been incomprehensible (less fluently processable), boring and thus less pleasant for non-experts. The latter experienced the presented architectural objects as more aggressive (compared to experts), which has been interpreted as representing higher negative arousal resulting from a lack of competence and safety feeling. Linking those considerations with the results found by Gifford et al. (2000), it is interesting, that the latter found no relation between arousal ratings and general assessments of architectural quality and pleasure experience.

Familiarity with, prototypicality and simplicity of architectural objects as influencing factors on automatic aesthetic evaluation (via effecting processing fluency) have been discussed as well by Mastandrea et al. (2011). Using the IAT, they observed shorter reaction times when non-expert participants were asked to associate positive words with classical architecture (compared to longer reaction times when associating with contemporary architecture). This response pattern corresponded with the one assessed for explicit evaluations for both architectural styles. Note that this correlation has not been found for the other stimulus type, i.e., figurative and abstract artworks. The authors concluded that, on the individual level, participants might have shown the same evaluative trend in both implicit and explicit aesthetic evaluation tasks because variety of stimuli within each architectural category is relatively low (which is not the case regarding art categories).

One physical feature which has always been regarded as important for architectural planning and designing (Le Corbusier, 1927) and which has been shown to influence emotional reactions (e.g., Hevner, 1935) and aesthetic preferences (e.g., Leder and Carbon, 2005; Bar and Neta, 2006) is contour, especially its amount of curvature. In one of the first fMRI studies on the influence of physical features of the built environment on brain activation, Vartanian et al. (2013) demonstrated that the effect of contour on beauty judgments can be extended to architecture. In their study non-expert participants showed beauty preferences for curvilinear over rectilinear spaces and on behavioral level this effect has been shown to be likely driven by pleasantness. Moreover, contemplating beauty of curvilinear spaces was associated exclusively with increased activity of the anterior cingulate cortex (ACC) over beauty judgments of rectilinear spaces. It has been demonstrated that the ACC contributes to reward and emotional processing (Kringelbach and Rolls, 2004; Liu et al., 2011). Taking together behavioral and neural results, Vartanian et al. (2013) conclude that aesthetic preferences for curvilinear contour is underpinned by reward and emotion, once again pointing to the role of emotion in aesthetic experience.

In this 2013 study, stimuli were controlled for the variables height and openness/enclosure because of some empirical results hinting at their influence on cognition and emotion when contemplating architecture (Franz et al., 2005; Meyers-Levy and Zhu, 2007). In a later study, the authors investigated the effects of those variables on aesthetic judgments, again by assessing behavioral and fMRI-data (Vartanian et al., 2015). Their results showed that higher ceilings and open rooms were more likely to be judged as more beautiful. Increased activity in frontal and parietal structures of the dorsal stream associated with visuospatial exploration and attention have been demonstrated for higher ceilings. Moreover, the authors suggested that involvement of the temporal lobes when judging the beauty of open rooms might be linked to their contribution to the temporal dynamics of visual motion representation.

Having focused on the aesthetics of buildings and their interior so far, it remains to address the influence of the context in which those buildings are beheld. With regard to the aesthetics of urban landscapes, it has been shown that urban scenes that constitute places with panoramic views, settings with historical/cultural significance and recreational places were considered as most attractive, whereas industrial and housing areas were rated as most unattractive (Galindo and Hidalgo, 2005). However, those categorical results were derived from listings of the most and least attractive places of one city (Málaga, Spain). The participants, who were residents who indicated a high sense of belonging for the city, also evaluated the restorative properties and the environmental characteristics of their listed places. The authors based the formation of the above listed urban aesthetic categories on two dimensions, that is the function and historical value of a scene, which they consider as important influencing factors regarding urban scene preferences. Findings by Van den Berg et al. (2003) and Galindo and Hidalgo (2005) further support the idea that the restorative capacity of a scene seems to be one of the criteria underlying environmental aesthetic preferences (Purcell et al., 2001).

Even more interesting than assessing preferences for rather broad urban aesthetic scenes is the question, to which visual, spatial properties those aesthetic preferences can be attributed. Weber et al. (2008) were narrowing down this issue by assessing the relationship between aesthetic judgments and selected visuospatial properties of complex streetscapes sampled from an existing mid-sized German town. Vegetation, stylistic uniformity, homogeneity of scale, and symmetry were observed as important factors. Corresponding to Gestalt psychology’s figure formation characteristics (later adapted for 3D architectural spaces by Weber, 1995) the results of participants’ beauty ratings suggested visual regularity as primary factor in beauty judgment. Precisely, factors such as symmetry and uniformity of lateral spatial boundaries of the streetscapes depicted in the photographs were influencing aesthetic judgments especially if those boundaries were formed by vegetation or by building types featuring stylistic uniformity. Surprisingly, the study revealed no significant differences between aesthetic judgments of experts and laymen.

#### Design

Nowadays a lot of entities can be “designed” in some way. In this section, we will shortly look at the few research findings addressing the more traditional fields of design. Empirical accounts indicate that, regarding the aesthetic appreciation of objects, usability of those objects or products is positively affected by visual aesthetics, but that this relation is mediated by an overall evaluation of “goodness” of the respective object (for a review, see e.g., Hassenzahl and Monk, 2010). Individual differences of aesthetic preferences for wall color of a domestic interior, which was furnished in one of three styles, was found by Whitfield (1984). An examination of the preferences for pieces of furniture, which should be also categorized according to those three styles, showed once more, that prototypical or familiar objects were preferred (Whitfield and Slatter, 1979). An influence of attitudes on interior design preferences has been demonstrated by Ritterfeld (2002). However, this effect has been solely shown to arise from social heuristic processing when the social information of the object (a piece of furniture) was decodable or consistent. This way of processing is thought to be more automatic and less controlled. In contrast, systematic analysis of the object’s structural, non-social properties appeared when social information was not decodable or inconsistent. Thus, failure of the social heuristic resulted in an increase of preference judgment latencies and a decrease of evaluation certainty. Even so, taking into account motivational factors, an automatic application of social heuristic processing can be overcome when accuracy or directional goals are present. To explain everyday aesthetic experiences and preferences, Ritterfeld (2002) proposed an integrative model comprising both target properties and motivational aspects as determining factors. Another study that also used chairs (classic vs. modern) as target design objects, investigated whether expertise in industrial design would influence preferences for the different styles on an implicit and explicit level (Mastandrea and Maricchiolo, 2014). The results indicated an automatic influence of expertise on aesthetic evaluation of those objects, with laypeople showing no specific preference orientation compared to experts who preferred modern over classic chairs.

## Conclusion

We have examined aspects of variability and stability regarding the aesthetic experience and aesthetic preferences for different domains of aesthetic appreciation. Within this scope, we wanted to rivet on the role of sensitive periods for the development of aesthetic taste, as well as on the importance of cognitive schemata and specifically scripts for the aesthetic experience in various aesthetic domains.

Bearing in mind that all aesthetic experience is situated and processed by individuals whose ontological development was influenced by nature and nurture (e.g., Jacobsen, 2006, 2010a), the diverse empirical pattern obtained in the present analysis may not be surprising. People differ and aesthetic domains do as well. Although some preferred features appear to be deeply rooted in the visual modality, symmetry or green vegetation, the visual aesthetic domains appear to show the highest variability. While mixed accounts of variability and stability were found for the domains of music, language, and architecture. It is important to note, however, that the mosaic of available research is patchy. While notions on sensitive periods in the development of musical taste exist, comparable research for visual domains of aesthetic appreciation is scarce. In the same vein, comparisons of individual differences of aesthetic experience across domains are currently hardly possible.

We suggest that within ontogenesis of the human perceptual and conceptual system, crucial foundations for the capacity of aesthetic evaluation are laid. To date, research on sensitive (or even critical) periods regarding, for instance, the development of basic characteristics of human sensory processing as well as of higher-order processes relevant for aesthetic appreciation, is still somewhat limited. In order to link such relevant sensitive periods to periods which may be crucial for the development of aesthetic taste, further research is required. Therefore, it will be important to isolate even more those perceptual and cognitive processes (and relevant interactions) which play a crucial role for the formation of aesthetic evaluations. We also believe that calling some attention to the significance of cognitive schemata and scripts in experimental aesthetics may help to understand some empirical findings better and add to models of aesthetic processing in various domains.

## Author Contributions

TJ and SB conceived and wrote the manuscript.

## Conflict of Interest Statement

The authors declare that the research was conducted in the absence of any commercial or financial relationships that could be construed as a potential conflict of interest.
